# Bacterial cell‐to‐cell signaling promotes the evolution of resistance to parasitic bacteriophages

**DOI:** 10.1002/ece3.2818

**Published:** 2017-02-21

**Authors:** Pierre Moreau, Stephen P. Diggle, Ville‐Petri Friman

**Affiliations:** ^1^ Imperial College London, Silwood Park CampusAscotBerkshireUK; ^2^ School of Life SciencesCentre for Biomolecular SciencesUniversity of NottinghamNottinghamUK; ^3^ Department of BiologyThe University of YorkYorkUK

**Keywords:** bacteriophage, coevolution, evolution, parasitism, quorum sensing, resistance

## Abstract

The evolution of host–parasite interactions could be affected by intraspecies variation between different host and parasite genotypes. Here we studied how bacterial host cell‐to‐cell signaling affects the interaction with parasites using two bacteria‐specific viruses (bacteriophages) and the host bacterium *Pseudomonas aeruginosa* that communicates by secreting and responding to quorum sensing (QS) signal molecules. We found that a QS‐signaling proficient strain was able to evolve higher levels of resistance to phages during a short‐term selection experiment. This was unlikely driven by demographic effects (mutation supply and encounter rates), as nonsignaling strains reached higher population densities in the absence of phages in our selective environment. Instead, the evolved nonsignaling strains suffered relatively higher growth reduction in the absence of the phage, which could have constrained the phage resistance evolution. Complementation experiments with synthetic signal molecules showed that the *Pseudomonas* quinolone signal (PQS) improved the growth of nonsignaling bacteria in the presence of a phage, while the activation of *las* and *rhl* quorum sensing systems had no effect. Together, these results suggest that QS‐signaling can promote the evolution of phage resistance and that the loss of QS‐signaling could be costly in the presence of phages. Phage–bacteria interactions could therefore indirectly shape the evolution of intraspecies social interactions and PQS‐mediated virulence in *P. aeruginosa*.

## 1 Introduction

The evolution of host–parasite interactions is sensitive to the underlying characteristics of the coevolving host and parasite genotypes (Lively & Dybdahl, 2000; Sorci, Moller, & Boulinier, 1997; Thompson, 2005; Vrijenhoek, 1986). For example, co‐evolutionary dynamics can follow either an arms race or fluctuating selection dynamics depending on the interacting parasite species (Betts, Kaltz, & Hochberg, 2014), while considerable variation exists in host species sensitivity to a one given parasite species (Thompson, 2005). This is especially true with bacteria, where small differences between different genotypes can have large effects on fitness. This has been shown to be the case for bacterial species that use quorum sensing (QS) signaling to regulate a considerable part of their genome according to the signal concentration in the surrounding environment, which is often directly linked with the cell density of the local bacterial population (Darch, West, Winzer, & Diggle, 2012; Waters & Bassler, 2005).

Quorum sensing‐signaling has been shown to regulate various important bacterial functions. For example, in the opportunistic pathogen *Pseudomonas aeruginosa*, QS systems regulate the expression of 6%–10% of *P. aeruginosa* genes, including those encoding virulence factors such as hydrogen cyanide, the galactophilic lectin LecA, elastase, rhamnolipids, and others involved in protein secretion and chemotaxis (Schuster, Lostroh, Ogi, & Greenberg, 2003; Schuster, Sexton, Diggle, & Greenberg, 2013; Williams & Camara, 2009). In *P*. *aeruginosa*, QS involves three major QS‐signaling pathways: the *las* and *rhl* systems that utilize *N*‐acylhomoserine lactones (AHLs) and a *Pseudomonas* Quinolone Signal (PQS) system that uses 2‐alkyl‐4‐quinolones (AQs) as QS signal molecules (Williams & Camara, 2009). The *las* and *rhl* systems are linked such that LasR drives the expression of lasI as well as rhlR and rhlI (Latifi, Foglino, Tanaka, Williams, & Lazdunski, 1996), and PQS and the *las* and *rhl* systems have also been found to interact (Diggle et al., 2003; McKnight, Iglewski, & Pesci, 2000). As a result, *P*. *aeruginosa* QS‐signaling pathways are inter‐linked and activation of several systems can be required for full expression of certain “traits” such as bacterial virulence factors (Williams & Camara, 2009).

While some benefits of *P. aeruginosa* QS‐signaling are linked to bacterial social lives via access to public goods (such as growth‐limiting iron and nutrients) (Diggle, Griffin, Campbell, & West, 2007; Harrison & Buckling, 2009), some benefits are only beneficial for individual QS‐signaling bacteria (e.g., private goods (Dandekar, Chugani, & Greenberg, 2012; Darch et al., 2012; West, Winzer, Gardner, & Diggle, 2012)). Furthermore, QS‐signaling regulated genes have been shown to be beneficial in nonsocial ecological contexts, which suggest that selection by abiotic environment or interspecies interactions could drive the evolution of QS‐signaling. For example, it has been shown that protist predation can select for bacterial cooperative behavior because the traits connected to the anti‐predatory defences are regulated by QS‐signaling (Friman, Diggle, & Buckling, 2013; Jousset et al., 2009). Furthermore, competition with *Candida albicans* has been shown to inhibit bacterial iron acquisition and virulence, which are both driven by QS‐mediated gene expression (Lopez‐Medina et al., 2015), while in contrast, the presence of another bacterium, *Staphylococcus aureus*, has been shown to affect *P. aeruginosa* virulence, antibiotic tolerance, and growth depending on the functionality of *P. aeruginosa* QS‐signaling system (Korgaonkar, Trivedi, Rumbaugh, & Whiteley, 2013; Michelsen et al., 2014). Interestingly, recent findings also suggest that QS can plastically affect bacterial ability to resist phage parasites. For example, *Escherichia coli* QS genes help to protect against parasites via phage receptor‐mediated effects (Hoyland‐Kroghsbo, Maerkedahl, & Svenningsen, 2013), while QS‐signaling has been shown to affect the *Vibrio anguillarum* mode of phage resistance via density‐dependent gene expression (Tan, Svenningsen, & Middelboe, 2015). A recently published paper also suggests that a dysfunctional *las* system can confer evolutionary benefits for *P. aeruginosa* in the presence of both phages and bacterial competitors due to relatively lower pleiotropic costs of phage resistance (Mumford & Friman, 2016). However, the evolutionary effects of different QS systems on phage resistance evolution are unknown.

Here we tested directly how intraspecific variation in *P. aeruginosa* QS‐signaling affects its ability to evolve resistance to phage parasites. We used a QS‐signaling PAO1 wild‐type strain and QS‐defective mutants that differed in their ability to produce (signal‐negative; *lasI*,* rhlI*,* pqsA*) and respond (signal‐blind; *lasR*,* rhlR* and *pqsR*) to QS signals. All strains were evolved in the absence and presence of two different bacteriophages before determining the evolutionary changes in the levels of phage resistance. Additional experiments were conducted to study whether activation of different QS systems with exogenously added signal molecules affects bacterial growth in the presence of a phage.

## 2 Materials and Methods

### 2.1 Study species

Quorum sensing‐positive, cooperating PAO1 and QS‐defective, cheating *lasI*,* rhlI*,* pqsA* (signal‐negative), and *lasR*,* rhlR,* and *pqsR* (signal‐blind) *P. aeruginosa* strains were used in this study (Wilder, Diggle, & Schuster, 2011). The “signal‐negative” mutants do not produce signals but still respond, whereas “signal‐blind” mutants neither produce nor respond to extra‐cellular signals. Two phage species, PT7 and 14/1, were used as parasites (Merabishvili et al., 2007).

### 2.2 Selection experiment and evolutionary assays

Bacteria (initial density ~3.7 × 10^2^ cells/ml) were cultured in 96‐well plates in the absence and presence of phages (initial density ~2.3 × 10^2^ cells/ml) in 220 μl of KB media (Friman et al., 2013) for 48 hrs (*N* = 4, 37°C, without shaking). Bacterial densities were measured at 24‐hour intervals with a spectrophotometer (Biotek, OD 600 nm). After 48 hr, all populations were plated on KB plates and eight colonies per population isolated for evolutionary assays. Resistance evolution of alone‐evolved and phage‐evolved populations was compared by measuring the bacterial growth in the presence and absence of ancestral phages after 24 hr in liquid KB media (same experimental conditions as above). The effect of phage selection on bacterial growth was determined as the relative growth of phage‐evolved versus alone‐evolved bacterial selection lines in the absence of phages.

### 2.3 Complementation experiments with synthetic QS‐signal molecules

All signal‐negative (*lasI*,* rhlI*,* pqsA*) and signal‐blind (*lasR*,* rhlR,* and *pqsR*) mutants were cultured in the absence and presence of PT7 phage and external signals in 220 μl of 1.5% CAA media (6.8 g/L of Na_2_HPO_4_, 3 g/L of KH_2_PO_4_, 0.5 g of NaCl/L, and 2.5 g of Casamino acids/L) for 8 days (20% of old population transferred to fresh media every 24 hr). The QS‐signaling of *lasI*,* rhlI*,* pqsA* mutants was activated using 50 μmol/L final concentrations of 3O‐C_12_‐HSL (*lasI*), C4‐HSL (*rhlI*), and PQS (*pqsA*) signal molecules (Diggle et al., 2007). Signal‐blind mutants were used as controls. All treatments were replicated four times and bacterial densities measured at 24‐hour intervals with a spectrophotometer (Biotek).

### 2.4 Statistical analysis

All data were analyzed with linear mixed models, and repeated measures analysis was used for the time dynamics data. Colony replicates were nested under population replicates in evolutionary assays. Bacterial OD data were log‐transformed before the analyses.

## 3 Results

### 3.1 Phage effects on QS‐signaling and nonsignaling bacteria

**Figure 1:**
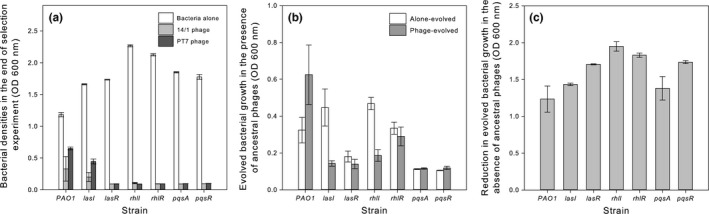
Panel (a) shows the densities of quorum sensing (QS)‐signaling and nonsignaling strains in the end of the selection experiment. White bars denote for bacteria‐alone, and gray bars bacteria‐phage treatments, respectively. Panel (b) shows the growth of alone‐evolved (white bars) and phage‐evolved (gray bars) bacterial clones in the presence of ancestral phage strains (data pooled over both phage species). Panel (c) shows the reduction in evolved bacterial strains’ growth in the absence of phages after the selection experiment (data pooled over both phage species) In all panels, bars show ±1 *SEM*

In bacterial monocultures, the QS‐signaling strain reached lower population densities in the absence of phages but higher population densities in the presence of phage compared to nonsignaling strains (signal × phage: *F*_1,80_ = 75.7, *p* < .001; *p* < .05 for all pairwise comparison; Figure 1a). Phages more clearly reduced the densities of nonsignaling strains and while the phage species had similar effects, phage 14/1 reduced the bacterial densities more in general (phage treatment: *F*_1,80_ = 729.9, *p* < .001; Figure 1a). Different QS mutants also grew differently: while *rhl* mutants reached highest population densities in the absence of phages, the *lasI* mutant reached a higher population density in the presence of phages compared to other QS‐defective mutants (phage treatment × bacterial strain: *F*_12,63_ = 36.8, *p* < .001).

We next compared the bacterial resistance evolution to ancestral phages between different treatments by growing isolated evolved clones in the presence of ancestral phages. We found that phage resistance evolved differently depending on the QS‐signaling ability (evolutionary history × QS functionality: *F*_1,160_ = 192.9, *p* < .001; Figure 1b). Specifically, the phage‐evolved QS‐signaling strain could grow better in the presence of ancestral phage compared to alone‐evolved QS‐signaling strains (Figure 1b). While the phage‐evolved nonsignaling strains grew generally worse compared to alone‐evolved nonsignaling strains (QS functionality: *F*_3,152_ = 19.8, *p* < .001), the strains with an impaired PQS QS system grew slightly better in the presence of ancestral phages if they had been evolving in the presence of phages during the selection experiment (evolutionary history × QS system: *F*_3,152_ = 19.7, *p* < .001; the effect of phage history within evolved *pqs* strains: *F*_1,40_ = 4.1, *p* < .001, no difference between *pqsA* and *pqsR* mutants (*p* > .05), Figure 1b). Both phages had similar effects for the resistance evolution with QS‐signaling and nonsignaling strains (phage species: *F*_1,159_ = 1.3, *p* = .249; data not shown). Interestingly, phage selection led to reduction in bacterial growth in the absence of phage in general (evolutionary history: *F*_1,48_ = 417, *p* < .001), while this reduction was relatively larger for nonsignaling strains (QS‐signaling: *F*_1,26_ = 4.3, *p* = .048; the effect of QS system nonsignificant: *F*_2,21_ = 3.1, *p* = .07, Figure 1c).

### 3.2 Effects of QS‐signaling activation for bacterial fitness in the presence of phages

**Figure 2:**
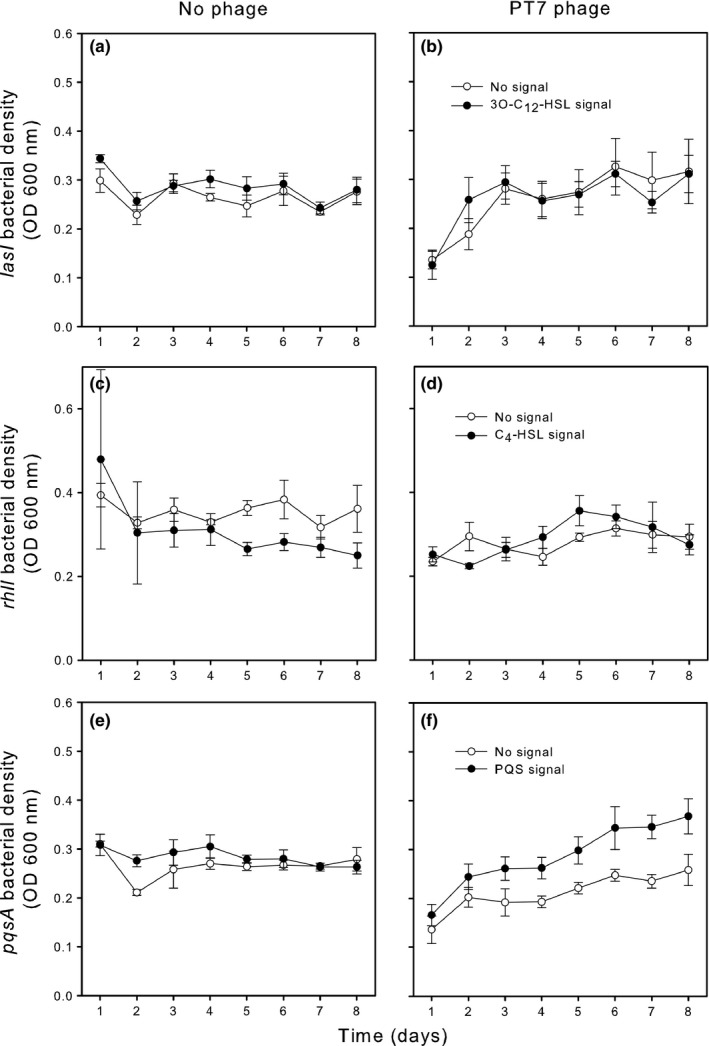
The effect of exogenously supplied signal on signal‐negative bacterial densities in the absence and presence of phage PT7. Panels (a) and (b) show the signal (black symbols) effect for *las*, panels (c) and (d) for *rhl* and panels (e) and (f) for *pqs* strains in the absence and presence of the PT7 phage. Bars show ±1 *SEM*

Phages reduced the densities of both signal‐blind (*F*_1,42.9_ = 32.987, *p* < .001) and signal‐negative strains (*F*_1,48_ = 10.740, *p* = .002; approximately from 0.3 to 0.15 at OD 600 nm; Figures 2 and S1). Expectedly, external signal had no effect on signal‐blind strains in the absence or presence of phages (signal: *F*_1,42.9_ = 0.95, *p* = .335; phage × signal: *F*_1,42.9_ = 0.054, *p* = .817, Fig. S1). However, external signal increased the density of the *pqsA* signal‐negative strain in the presence of a phage (phage × signal: *F*_1,48_ = 7.724, *p* = .008), while no effect was observed with signal‐negative *lasI* and *rhlI* strains (phage × strain × signal: *F*_2,48_ = 5.916, *p* = .005, Figure 2). None of the signals affected the densities of nonsignaling bacteria in the absence of phages (Figure 2). Together, these results suggest that activation of the alkyl‐quinolone QS system by PQS increases the ability of *P. aeruginosa* to grow in the presence of phage.

## 4 Discussion

Here we studied experimentally how intraspecific variation in *P. aeruginosa* QS‐signaling affects its ability to evolve resistance to phage parasites. We found that a QS‐signaling‐positive strain evolved higher levels of phage resistance during a short‐term selection experiment. This difference was unlikely to be driven by demographic effects as the nonsignaling strains reached generally higher population densities in the absence of phages—resistance mutations arise generally faster in large populations due high mutation supply rates and frequent host–parasite encounter rates (Lopez‐Pascua & Buckling, 2008). However, we found that phage selection led to relatively larger reduction in the growth of nonsignaling versus signaling strains and that the activation of the alkyl‐quinolone (AQ) QS system by PQS, promoted bacterial growth in the presence of phage. Together, these results suggest that potential phage resistance mutations were costly and under AQ QS‐regulation.

The addition of exogenous QS signals did not affect the growth of *lasI* and *rhlI* strains in the absence or presence of phage (both signal‐negative and signal‐blind). This suggests that activation of acyl‐homoserine lactone QS systems unlikely affected the evolution of phage resistance via population density‐mediated demographic effects (Lopez‐Pascua & Buckling, 2008). The lack of signal effect can be most likely attributed to the experimental environment, which did not favor QS‐signaling: Resources are readily available in KB media (Friman et al., 2013) and the QS expression thus unlikely offered any additional benefits for the signaling bacteria. It is known that knocking out the *las* system will have downstream effects on the *rhl* system (Latifi et al., 1996), and as a result, it is often thought that the *las* mutants behave similarly as the *rhl* mutants. However, it has also been shown that *las* and *rhl* systems can be decoupled and that the interactions between different QS systems depend on the environmental conditions (Diggle et al., 2003). As a result, the role of acyl‐homoserine lactone QS systems for the phage resistance evolution should be explored in different environmental contexts in the future.

Our findings partly contrast and support the previously published results. For example, it has been previously found that the activation of the *lasI* and *rhlI* QS systems can increase *P. aeruginosa* susceptibility to pilus‐specific phages (Glessner, Smith, Iglewski, & Robinson, 1999). Even though the PT7 phage receptor is not known (Friman et al., 2016), our data suggest that it might not use pilus as its receptor as no increase in *las* and *rhl* mutants’ phage susceptibility was found. However, our short‐term results support a previous long‐term study where no difference was found in the levels of resistance evolution between PAO1 and *lasR* mutant strains under PT7 phage selection (Mumford & Friman, 2016). However, it was observed that *lasR* mutant strain could evolve higher levels of resistance in the presence of other competing bacteria (Mumford & Friman, 2016). This suggests that *las* system could affect phage resistance evolution on a longer timescale in multispecies microbial communities. Mechanistically, this difference could be at least partly explained by relatively lower cost of resistance of the *lasR* strain (Mumford & Friman, 2016). Similarly, we found that that phage‐evolved QS‐signaling and nonsignaling strains suffered from reduced growth in the absence of phages indicative of cost of resistance (Figure 1c). Crucially, the magnitude of the cost was relatively higher for the nonsignaling strains, which could partly explain the lowered rate of resistance evolution of the nonsignaling strain. Together, these results suggest that fully functional *las* and *rhl* QS systems were needed for the rapid evolution of phage resistance in our study system.

Similar to *las* and *rhl* QS systems, PQS signal had no effect on the growth of signal‐negative *pqsA* or signal‐blind *pqsR* strains in the absence of phage. This suggests that the benefit of PQS signal for bacterial growth was not driven by demographic effects. While it is possible that QS signals can mediate phage resistance plastically via signal‐mediated expression of phage receptors (Hoyland‐Kroghsbo et al., 2013), we found that addition of PQS signals did not confer immediate growth benefit for the *pqsA* mutant (no difference after 1 day growth, Figure 2). Instead, exogenously supplied PQS signal increased the growth of the nonsignaling *pqsA* mutant growth in the presence of phage only after few serial transfers. Even though we did not directly measure the changes in bacterial resistance to phages in the end of the signal addition experiment, these data suggest that activation of *pqs* system likely conferred an evolutionary benefit for the *pqsA* mutant. Interestingly, we also found that *pqs* mutants evolved very low levels of resistance during the first selection experiment even in the absence of exogenous signal. This could be partly explained by the relatively weak phage‐mediated growth reduction experienced by the *pqsA* strain. Even though it is difficult to make direct comparisons between our two experiments due to different culture conditions and timescales, our results suggest that the PQS system could be important for the phage resistance evolution via two mechanisms: by directly activating the phage resistance genes or by ameliorating the costs of resistance via a defective PQS system.

The link between PQS and phage resistance is also supported by other recent studies. For example, phage resistance evolution has been linked with the overexpression of QS‐signaling regulated pyocyanin and pyoverdine production in *P. aeruginosa* (Hosseinidoust, Tufenkji, & van de Ven, 2013). Moreover, it has been demonstrated that 14/1 phage attack can directly increase the production of PQS with PAO1, which suggest that quinolones play an important role for bacterial survival in the presence of phage parasites (De Smet et al., 2016). However, also the opposite pattern has also been found where phage selection has been shown to lead to a loss of QS by favoring mutations at the *mvfR* and *lasR* loci (Davies et al., 2016). A crucial difference between these experiments was that Davies et al. (2016) used lysogenic phages while this and previous studies by Hosseinidoust et al. (2013) and De Smet et al. (2016) used lytic phages belonging to a family *Myoviridae*. A recently published paper has also demonstrated that lysogenic phages can turn a susceptible *E. coli* strain resistant to a lytic phage via CRISPR‐Cas system modification (Yosef, Manor, Kiro, & Qimron, 2015). It could be that lytic and lysogenic phages oppose contrasting selection pressures on *P. aeruginosa* QS‐signaling systems via phage resistance evolution—a hypothesis that will be tested in the future.

Our results have potential clinical relevance because QS is a key regulator of important virulence factors in *P. aeruginosa*. First, it is possible that phage selection might increase the proportion of virulent *P. aeruginosa* genotypes both in the natural and clinical environments via positive selection for functional PQS QS system. While it has been shown that lysogenic phages play important role in the context of lung infections of patients with cystic fibrosis (James et al., 2015), the significance of lytic phages is relatively unknown. Potential conflicts with the host‐associated lysogenic phages might lead to unexpected treatment outcomes, and care should be taken to select lytic phage species that do not favor virulent QS‐signaling strains. Furthermore, if phage resistance is positively correlated with functional QS systems, phage therapy could be potentially combined with antivirulence strategies that impair bacterial QS (Allen, Popat, Diggle, & Brown, 2014). Lastly, our results suggest that phage selection might indirectly affect the evolution of social interactions with bacteria by potentially changing the relative benefit of cooperation and cheating. Multilevel selection acting on bacterial cell‐to‐cell signaling is thus likely important factor for explaining high *P. aeruginosa* intraspecies diversity.

## Conflict of Interest

None declared.

## References

[ece32818-bib-0001] Allen, R. C. , Popat, R. , Diggle, S. P. , & Brown, S. P. (2014). Targeting virulence: Can we make evolution‐proof drugs? Nature Reviews Microbiology, 12, 300–308. 24625893 10.1038/nrmicro3232

[ece32818-bib-0002] Betts, A. , Kaltz, O. , & Hochberg, M. E. (2014). Contrasted coevolutionary dynamics between a bacterial pathogen and its bacteriophages. Proceedings of the National Academy of Sciences of the United States of America, 111, 11109–11114. 25024215 10.1073/pnas.1406763111 PMC4121802

[ece32818-bib-0003] Dandekar, A. A. , Chugani, S. , & Greenberg, E. P. (2012). Bacterial quorum sensing and metabolic incentives to cooperate. Science, 338, 264–266. 23066081 10.1126/science.1227289 PMC3587168

[ece32818-bib-0004] Darch, S. E. , West, S. A. , Winzer, K. , & Diggle, S. P. (2012). Density‐dependent fitness benefits in quorum‐sensing bacterial populations. Proceedings of the National Academy of Sciences of the United States of America, 109, 8259–8263. 22566647 10.1073/pnas.1118131109 PMC3361460

[ece32818-bib-0005] Davies, E. V. , James, C. E. , Williams, D. , O'Brien, S. , Fothergill, J. L. , Haldenby, S. , et al. (2016). Temperate phages both mediate and drive adaptive evolution in pathogen biofilms. Proceedings of the National Academy of Sciences of the United States of America, 113, 8266–8271. 27382184 10.1073/pnas.1520056113 PMC4961188

[ece32818-bib-0006] De Smet, J. , Zimmermann, M. , Kogadeeva, M. , Ceyssens, P. J. , Vermaelen, W. , Blasdel, B. , … Lavigne, R. (2016). High coverage metabolomics analysis reveals phage‐specific alterations to *Pseudomonas aeruginosa* physiology during infection. ISME Journal, 10, 1823–1835. 26882266 10.1038/ismej.2016.3 PMC5029163

[ece32818-bib-0007] Diggle, S. P. , Griffin, A. S. , Campbell, G. S. , & West, S. A. (2007). Cooperation and conflict in quorum‐sensing bacterial populations. Nature, 450, 411–414. 18004383 10.1038/nature06279

[ece32818-bib-0008] Diggle, S. P. , Winzer, K. , Chhabra, S. R. , Worrall, K. E. , Camara, M. , & Williams, P. (2003). The *Pseudomonas aeruginosa* quinolone signal molecule overcomes the cell density‐dependency of the quorum sensing hierarchy, regulates rhl‐dependent genes at the onset of stationary phase and can be produced in the absence of LasR. Molecular Microbiology, 50, 29–43. 14507361 10.1046/j.1365-2958.2003.03672.x

[ece32818-bib-0009] Friman, V. P. , Diggle, S. P. , & Buckling, A. (2013). Protist predation can favour cooperation within bacterial species. Biology Letters, 9(5), p.20130548. 10.1098/rsbl.2013.0548 PMC3971697 23945212

[ece32818-bib-0010] Friman, V. P. , Soanes‐Brown, D. , Sierocinski, P. , Molin, S. , Johansen, H. K. , Merabishvili, M. , et al. (2016). Pre‐adapting parasitic phages to a pathogen leads to increased pathogen clearance and lowered resistance evolution with *Pseudomonas aeruginosa* cystic fibrosis bacterial isolates. Journal of Evolutionary Biology, 29, 188–198. 26476097 10.1111/jeb.12774

[ece32818-bib-0011] Glessner, A. , Smith, R. S. , Iglewski, B. H. , & Robinson, J. B. (1999). Roles of *Pseudomonas aeruginosa* las and rhl quorum‐sensing systems in control of twitching motility. Journal of Bacteriology, 181, 1623–1629. 10049396 10.1128/jb.181.5.1623-1629.1999 PMC93554

[ece32818-bib-0012] Harrison, F. , & Buckling, A. (2009). Cooperative production of siderophores by *Pseudomonas aeruginosa* . Frontiers in Bioscience, 14, 4113–4126. 10.2741/3516 19273338

[ece32818-bib-0013] Hosseinidoust, Z. , Tufenkji, N. , & van de Ven, T. G. M. (2013). Predation in homogeneous and heterogeneous phage environments affects virulence determinants of *Pseudomonas aeruginosa* . Applied and Environment Microbiology, 79, 2862–2871. 10.1128/AEM.03817-12 PMC3623153 23435883

[ece32818-bib-0014] Hoyland‐Kroghsbo, N. M. , Maerkedahl, R. B. , & Svenningsen, S. L. (2013). A quorum‐sensing‐induced bacteriophage defense mechanism. MBio, 4, e00362–00312. 10.1128/mBio.00362-12 PMC3624510 23422409

[ece32818-bib-0015] James, C. E. , Davies, E. V. , Fothergill, J. L. , Walshaw, M. J. , Beale, C. M. , Brockhurst, M. A. , et al. (2015). Lytic activity by temperate phages of *Pseudomonas aeruginosa* in long‐term cystic fibrosis chronic lung infections. ISME Journal, 9, 1391–1398. 25461970 10.1038/ismej.2014.223 PMC4351911

[ece32818-bib-0016] Jousset, A. , Rochat, L. , Pechy‐Tarr, M. , Keel, C. , Scheu, S. , & Bonkowski, M. (2009). Predators promote defence of rhizosphere bacterial populations by selective feeding on non‐toxic cheaters. The ISME Journal, 3, 666–674. 19322247 10.1038/ismej.2009.26

[ece32818-bib-0017] Korgaonkar, A. , Trivedi, U. , Rumbaugh, K. P. , & Whiteley, M. (2013). Community surveillance enhances *Pseudomonas aeruginosa* virulence during polymicrobial infection. Proceedings of the National Academy of Sciences of the United States of America, 110, 1059–1064. 23277552 10.1073/pnas.1214550110 PMC3549110

[ece32818-bib-0018] Latifi, A. , Foglino, M. , Tanaka, K. , Williams, P. , & Lazdunski, A. (1996). A hierarchical quorum‐sensing cascade in *Pseudomonas aeruginosa* links the transcriptional activators LasR and RhIR (VsmR) to expression of the stationary‐phase sigma factor RpoS. Molecular Microbiology, 21, 1137–1146. 8898383 10.1046/j.1365-2958.1996.00063.x

[ece32818-bib-0019] Lively, C. M. , & Dybdahl, M. F. (2000). Parasite adaptation to locally common host genotypes. Nature, 405, 679–681. 10864323 10.1038/35015069

[ece32818-bib-0020] Lopez‐Medina, E. , Fan, D. , Coughlin, L. A. , Ho, E. X. , Lamont, I. L. , Reimmann, C. , … Koh, A. Y. (2015). *Candida albicans* inhibits *Pseudomonas aeruginosa* virulence through suppression of pyochelin and pyoverdine biosynthesis. Plos Pathogens, 11(8), p.e1005129. 10.1371/journal.ppat.1005129 PMC4552174 26313907

[ece32818-bib-0021] Lopez‐Pascua, L. C. , & Buckling, A. (2008). Increasing productivity accelerates host‐parasite coevolution. Journal of Evolutionary Biology, 21, 853–860. 18284514 10.1111/j.1420-9101.2008.01501.x

[ece32818-bib-0022] McKnight, S. L. , Iglewski, B. H. , & Pesci, E. C. (2000). The *Pseudomonas* quinolone signal regulates rhl quorum sensing in *Pseudomonas aeruginosa* . Journal of Bacteriology, 182, 2702–2708. 10781536 10.1128/jb.182.10.2702-2708.2000 PMC101972

[ece32818-bib-0023] Merabishvili, M. , Verhelst, R. , Glonti, T. , Chanishvili, N. , Krylov, V. , Cuvelier, C. , et al. (2007). Digitized fluorescent RFLP analysis (fRFLP) as a universal method for comparing genomes of culturable dsDNA viruses: Application to bacteriophages. Research in Microbiology, 158, 572–581. 17719750 10.1016/j.resmic.2007.06.002

[ece32818-bib-0024] Michelsen, C. F. , Christensen, A. M. , Bojer, M. S. , Hoiby, N. , Ingmer, H. , & Jelsbak, L. (2014). *Staphylococcus aureus* alters growth activity, autolysis, and antibiotic tolerance in a human host‐adapted *Pseudomonas aeruginosa* lineage. Journal of Bacteriology, 196, 3903–3911. 25182495 10.1128/JB.02006-14 PMC4248816

[ece32818-bib-0025] Mumford, R. , & Friman, V. P. (2016). Bacterial competition and quorum‐sensing signalling shapes the eco‐evolutionary outcomes of model in vitro phage therapy. Evolutionary Applications, 10, 161–169. 28127392 10.1111/eva.12435 PMC5253424

[ece32818-bib-0026] Schuster, M. , Lostroh, C. P. , Ogi, T. , & Greenberg, E. P. (2003). Identification, timing, and signal specificity of *Pseudomonas aeruginosa* quorum‐controlled genes: A transcriptome analysis. Journal of Bacteriology, 185, 2066–2079. 12644476 10.1128/JB.185.7.2066-2079.2003 PMC151497

[ece32818-bib-0027] Schuster, M. , Sexton, D. J. , Diggle, S. P. , & Greenberg, E. P. (2013). Acyl‐homoserine lactone quorum sensing: From evolution to application. Annual Review of Microbiology, 67, 43–63. 10.1146/annurev-micro-092412-155635 23682605

[ece32818-bib-0028] Sorci, G. , Moller, A. P. , & Boulinier, T. (1997). Genetics of host‐parasite interactions. Trends in Ecology & Evolution, 12, 196–200. 21238038 10.1016/s0169-5347(97)01056-2

[ece32818-bib-0029] Tan, D. , Svenningsen, S. L. , & Middelboe, M. (2015). Quorum sensing determines the choice of antiphage defense strategy in vibrio anguillarum. MBio, 6, e00627. 26081633 10.1128/mBio.00627-15 PMC4471561

[ece32818-bib-0030] Thompson, J. N. (2005). The geographic mosaic of coevolution. Chicago, IL: University of Chicago Press.

[ece32818-bib-0031] Vrijenhoek, R. C. (1986). Host‐parasite coevolution: Ecology and genetics of host‐parasite interactions. Science, 232, 112. 10.1126/science.232.4746.112 17774013

[ece32818-bib-0032] Waters, C. M. , & Bassler, B. L. (2005). Quorum sensing: Cell‐to‐cell communication in bacteria. Annual Review of Cell and Developmental Biology, 21, 319–346. 10.1146/annurev.cellbio.21.012704.131001 16212498

[ece32818-bib-0033] West, S. A. , Winzer, K. , Gardner, A. , & Diggle, S. P. (2012). Quorum sensing and the confusion about diffusion. Trends in Microbiology, 20, 586–594. 23084573 10.1016/j.tim.2012.09.004

[ece32818-bib-0034] Wilder, C. N. , Diggle, S. P. , & Schuster, M. (2011). Cooperation and cheating in *Pseudomonas aeruginosa*: The roles of the las, rhl and pqs quorum‐sensing systems. The ISME Journal, 5, 1332–1343. 21368905 10.1038/ismej.2011.13 PMC3146268

[ece32818-bib-0035] Williams, P. , & Camara, M. (2009). Quorum sensing and environmental adaptation in *Pseudomonas aeruginosa*: A tale of regulatory networks and multifunctional signal molecules. Current Opinion in Microbiology, 12, 182–191. 19249239 10.1016/j.mib.2009.01.005

[ece32818-bib-0036] Yosef, I. , Manor, M. , Kiro, R. , & Qimron, U. (2015). Temperate and lytic bacteriophages programmed to sensitize and kill antibiotic‐resistant bacteria. Proceedings of the National Academy of Sciences of the United States of America, 112, 7267–7272. 26060300 10.1073/pnas.1500107112 PMC4466736

