# Supplementary material for: Bacterial cell‐to‐cell signaling promotes the evolution of resistance to parasitic bacteriophages
Source: Ecol Evol. 2017 Feb 21;7(6):1936–41. doi: 10.1002/ece3.2818 (PMC5355186; doi:10.1002/ece3.2818)
Supplement: Supplementary file 1 [file ECE3-7-1936-s001.docx]

**Supplementary information**

**

**

**Supplementary figure 1.** The effect of exogenously supplied signal on signal-blind bacterial densities in the absence and presence of phage. Panels (a) and (b) show signal (black symbols) effect for *las*, panels (c) and (d) for *rhl* and panels (e) and (f) for *pqs* strains in the absence and presence of PT7 phage. Bars show ±1 s.e.m.
